# Growth Hormone-Releasing Peptide-6 (GHRP-6) Ameliorates Post-Infarct Ventricular Remodeling and Systolic Dysfunction in a Model of Permanent Coronary Ligation

**DOI:** 10.3390/ph19030468

**Published:** 2026-03-12

**Authors:** Linlin Wang, Arielis Rodriguez-Ulloa, Jorge Berlanga-Acosta, Ariana García-Ojalvo, Angel Abreu-Cruz, Luis Javier Gonzalez-López, Vladimir Besada-López, Yassel Ramos-Gómez, Gerardo Guillén-Nieto, Baohong Jiang

**Affiliations:** 1 Shanghai Institute of Materia Medica, Chinese Academy of Sciences, Shanghai 201203, China; wanglinlin@simm.ac.cn; 2 Center for Genetic Engineering and Biotechnology, Havana 10660, Cuba; arielis.rodriguez@cigb.edu.cu (A.R.-U.); luis.javier@cigb.edu.cu (L.J.G.-L.); vladimir.besada@cigb.edu.cu (V.B.-L.); yassel.ramos@cigb.edu.cu (Y.R.-G.); gerardo.guillen@cigb.edu.cu (G.G.-N.); 3 Department of Cardiology, Center for Surgical and Medical Research, Havana 10400, Cuba; aabreu@infomed.sld.cu; 4 China-Cuba Biotechnology Joint Innovation Center, Yongzhou 425000, China

**Keywords:** GHRP-6, heart infarction, systolic dysfunction, ejection fraction

## Abstract

**Background/Objective**: GHRP-6 is a GH secretagogue hexapeptide with expanding and promising cardioprotective effects. Having determined 0.4 mg/kg as the minimum effective dose for enhancing inotropy based on echocardiographic parameters in healthy rats, we implemented a non-reperfusion myocardial infarct model, with its consequent left ventricle wall thinning and ballooning, via permanent left descending coronary artery ligation. **Methods**: Rats were assigned to three groups: sham-operated/normal rats, infarcted + saline-treated control rats, and infarcted + GHRP-6-administration rats. Treatments were initiated post-surgery and continued for 7 days. On day 7, the animals were echocardiographically and histologically evaluated. For mitochondrial proteomic analysis, an additional 12 healthy rats were used. Six animals received GHRP-6 or normal saline and were observed for 6 h after the inoculation. **Results**: Here, we show that GHRP-6 attenuated myocardial tissue demise, reduced myocardial interstitial fibrosis/scarring, and integrally improved left ventricle physiology. The proteomic analysis indicated that the GHRP-6 cardioprotective effects may be theoretically mediated by the concerted upregulation of proteins/pathways involved in fatty acid beta-oxidation, apoptosis prevention pathways, antioxidant defenses, and mitochondrial metabolic reprogramming. **Conclusions**: GHRP-6 is a potent cardioprotective candidate attenuating morphological and functional outcomes caused by late ischemia.

## 1. Introduction

Although there has been a significant improvement in the management of acute myocardial infarction (AMI) over the past three decades, this condition remains a common medical emergency and a worldwide leading cause of morbidity and mortality [1]. Histopathologically, AMI is, by definition, a myocardial coagulative necrosis resulting from a sudden ischemic event [2], leading to mitochondrial functional collapse and subsequent ATP production deficit [3]. Early and successful myocardial reperfusion with the use of thrombolytic therapy or primary percutaneous coronary intervention (PCI) is the most effective strategy for reducing the size of the necrotic area, reducing hemodynamic instability, and improving the clinical outcome [4].

Despite timely PCI, AMI patients still experience significant acute myocardial functional complications [5]. Thus, preventing post-infarction ventricular failure is an important goal [6] to reduce the incidence of subsequent unfavorable outcomes, including cardiogenic shock and death [7]. Classic studies dated six decades ago indicated that the reperfusion itself accounts for half of the final infarct size and leads to subsequent myocardial dysfunction [8]. Consequently, cardioprotective strategies aimed at reducing infarct size and counteracting ventricular mechanical dysfunction are urgently needed [4]. Cardioprotection arises from the capacity of cardiac cells to activate or enhance endogenous survival signaling, enabling them to withstand lethal cellular stress [9].

Growth hormone releasing peptide-6 (GHRP-6), a synthetic hexapeptide (His-DTrp-Ala-Trp-DPhe-Lys-NH2) of low molecular weight, represents a key member of the GHRP family. Initially characterized for its potent growth hormone (GH)-releasing activity, the pharmacological profile of GHRP-6 has expanded considerably, with emerging evidence positioning it as a promising agent for cardiac protection [10]. Mechanistically, GHRP-6 functions as a ghrelin mimetic, exerting its effects through agonist activity at the growth hormone secretagogue receptor type 1a (GHSR1a) [11]. Additionally, it has been shown to interact with the extracellular domain of the cluster of differentiation 36 (CD36) receptor, implicating this pathway in its biological actions [12]. GHRP-6 has been shown to prevent and attenuate cardiac cell death and LV (left ventricle) failure in a variety of experimental scenarios [10,13,14,15]. A seminal study demonstrated that GHRP-6 administration prevented sudden death in a canine model of sudden cardiac death, where dilated cardiomyopathy (DCM) was induced by rapid cardiac pacing, followed by acute myocardial ischemia [16]. In the search for a mechanistic base, we induced AMI in pigs by occluding the left circumflex artery for one hour, followed by 4 days of reperfusion, in which GHRP-6 intervention reduced infarcted territory by 78%. While the study contributed to elucidating the mechanisms underpinning the GHRP-6 myocardial salvage effect, a major limitation is the lack of a post-infarct ventricular functional characterization [17]. Although GHRP family members have historically demonstrated cardioprotective effects [18,19,20], a variety and dispersion of dose ranges is observed. Having primarily established a GHRP-6 inotropic effective dose, based on echocardiographic parameters, we demonstrate here that therapeutic administrations of GHRP-6 translated into post-infarct systolic functional improvement, along with a biological meaningful reduction in myocardial tissue damage and interstitial scarring. Our mitochondrial proteomic study in normal hearts indicates that GHRP-6 may concertedly activate different proteins/pathways involved in bioenergetic homeostasis, myocardial metabolic plasticity, and pro-survival mechanisms.

While growth hormone secretagogue (GHS) family members like GHRP-6 have demonstrated cardioprotective effects in ischemia/reperfusion models, these studies primarily focused on acute cardiomyocyte death prevention. In contrast, our study is the first to systematically evaluate GHRP-6’s therapeutic potential in a permanent myocardial infarction model (characterized by sustained ventricular dilation and systolic dysfunction), demonstrating that subacute administration significantly improves left ventricular systolic function and inhibits adverse remodeling. Although our mitochondrial proteomic analysis was conducted in intact hearts, it further reveals that GHRP-6 may act through promoting cardiomyocytes survival pathways, optimizing fatty acid metabolism and mitochondrial metabolic plasticity, providing novel insights beyond those for classical anti-oxidant/anti-apoptotic mechanisms.

## 2. Results

### 2.1. GHRP-6 Doses: Acute Inotropic Response in Healthy Rats

**Figure 1:**
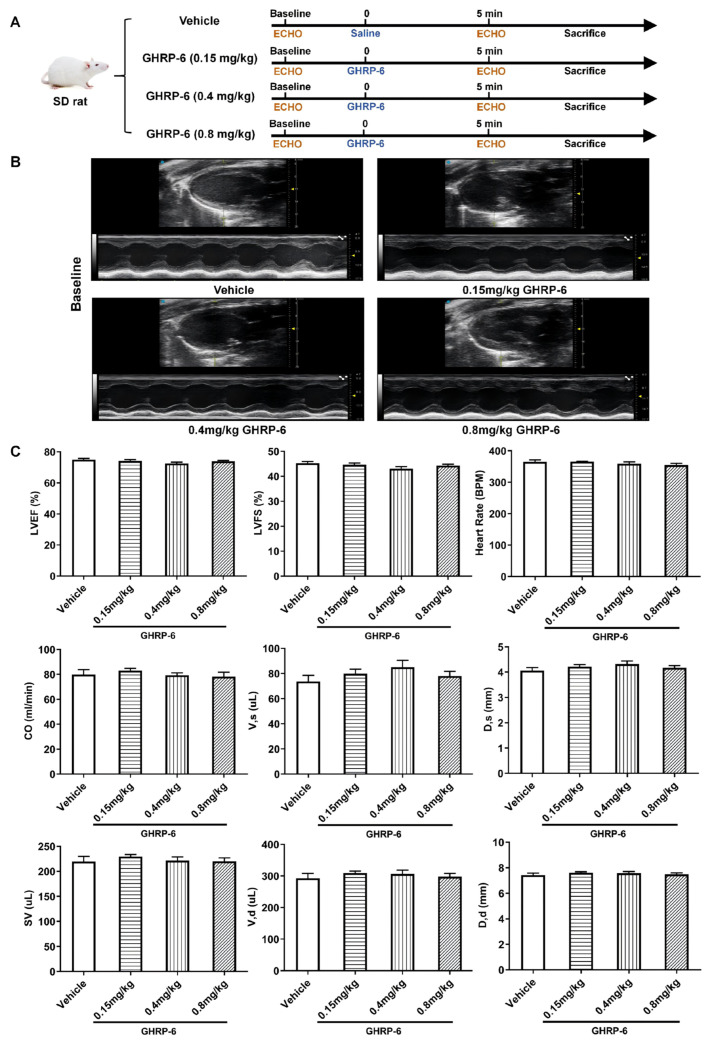
Baseline parameters of rats before GHRP-6 dose administration evaluated through transthoracic echocardiography. (**A**) Experimental flowchart. (**B**) Representative images of echocardiography data shown in B- and M-mode images. (**C**) Quantitative characterization of the ventricular structure and functional parameters. Data indicate that all the animals exhibited a normal ventricular morphology and physiology; *n* = 10 for each group. All the values were expressed as mean ± SEM. Diastolic dimension (D, d); systolic dimension (D, s); diastolic volume (V, d); systolic volume (V, s); left ventricular fractional shortening (LVFS); left ventricular ejection fraction (LVEF); cardiac output (CO); stroke volume (SV). Group differences were assessed using one-way ANOVA for continuous variables with homogeneity of variance and Brown–Forsythe and Welch’s ANOVA tests for heteroscedasticity data. Nonparametric analyses were performed using the Kruskal–Wallis test for data that do not meet normality.

The effect of GHRP-6 doses was characterized using structural and functional echocardiographic parameters. As shown in Figure 1, the baseline echocardiographic recordings indicated that all the selected myocardial structural and functional parameters were similar among all the groups.

**Figure 2:**
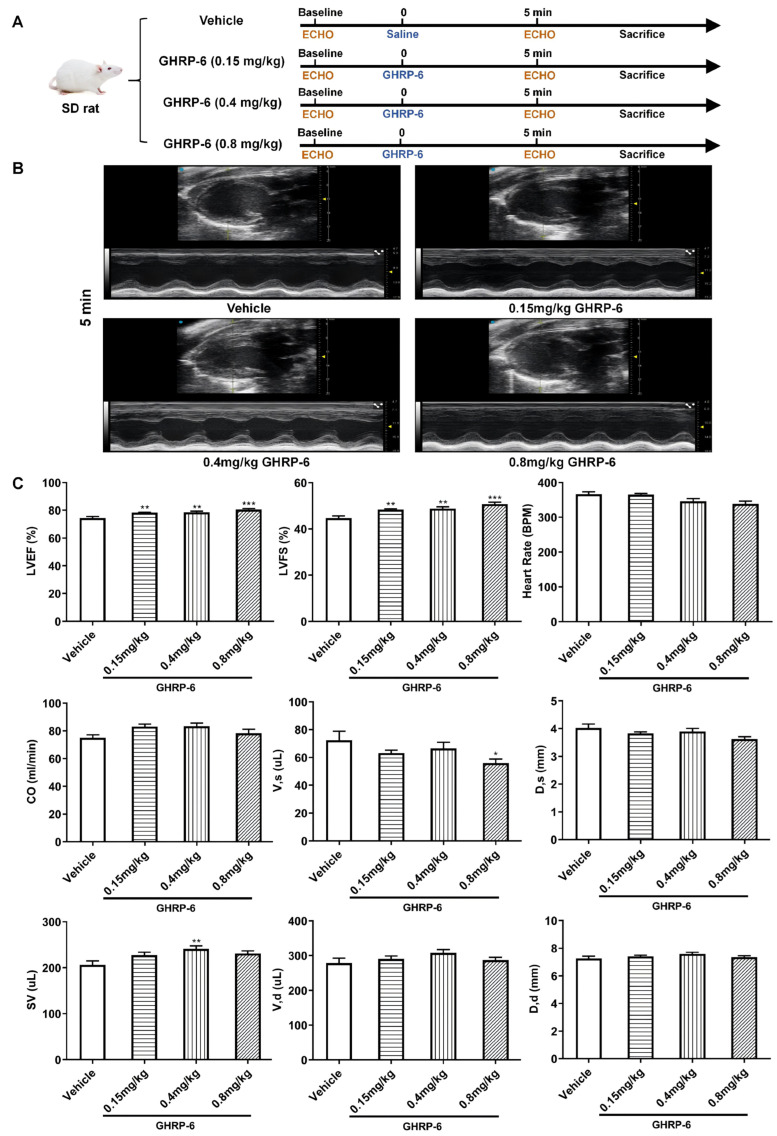
Ventricular structure and function 5 min after GHRP-6 dose administration, evaluated by transthoracic echocardiography. (**A**) Experimental flowchart. (**B**) Representative images of echocardiography data shown in B- and M-mode images. (**C**) Quantification of ventricular structure and function. Data indicate that GHRP-6 induces an acute inotropic response. All the values are expressed as mean ± SEM; *n* ≥ 9 for each group. * *p* < 0.05, ** *p* < 0.01, *** *p* < 0.001 versus vehicle results. Group differences were assessed using one-way ANOVA for continuous variables with homogeneity of variance and Brown–Forsythe and Welch’s ANOVA tests for heteroscedasticity data. Nonparametric analyses were performed using the Kruskal–Wallis test for data that do not meet normality.

The GHRP-6 dose–response assessment was performed on the basis of healthy hearts within physiological ranges. Five minutes after GHRP-6 administration, a significant increase in LVEF (78.3 ± 1.1% versus 74.4 ± 3.6%, *p* = 0.0084) and LVFS (48.3 ± 1.1% versus 44.7 ± 3.1%, *p* = 0.008) was observed in the group of rats receiving the lowest dose of 0.15 mg/kg as compared to saline vehicle group. A similar response was detected in the group of rats receiving GHRP-6 at 0.4 mg/kg. Correspondingly, significant increases in LVEF (78.6 ± 2.5% versus 74.4 ± 3.6%, *p* = 0.0055) and LVFS (48.8 ± 2.4% versus 44.7 ± 3.1%, *p* = 0.0037) were registered. Moreover, this dose level was the only dose that induced a significant increase in SV (241.4 ± 19.4 μL versus 206.1 ± 28.2 μL, *p* = 0.0081) in relation to that of the saline vehicle and the two other GHRP-6 dose levels studied. GHRP-6 administration at 0.8 mg/kg increased LVEF (80.5 ± 2.4% versus 74.4 ± 3.6%, *p* < 0.0001), and LVFS (50.7 ± 2.5% versus 44.7 ± 3.1%, *p* < 0.0001). Paradoxically, this dose induced the lowest systolic volume V, s levels (56.0 ± 9.2 μL versus 72.4 ± 20.4 μL, *p* = 0.0479) (Figure 2).

### 2.2. GHRP-6 Intervention Ameliorated Post-Infarct Ventricular Failure

**Figure 3:**
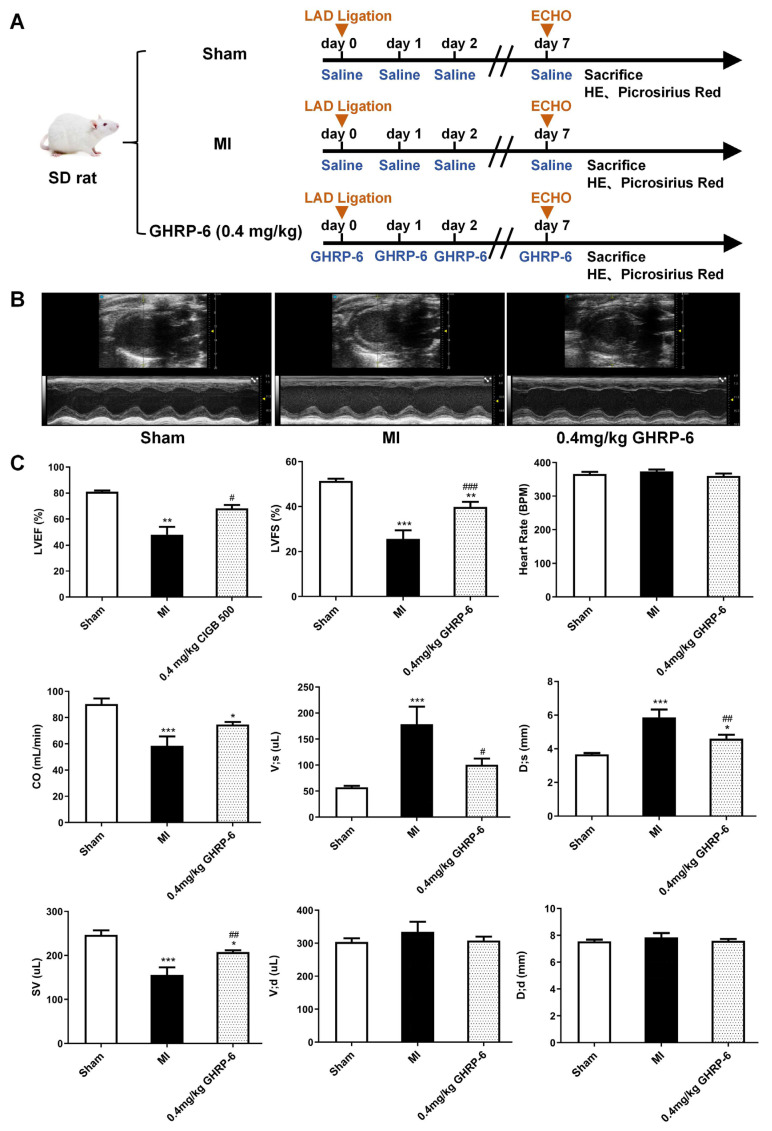
Post-infarct ventricular structural and functional parameters evaluated by echocardiography after 7th GHRP-6 administration. (**A**) Experimental flowchart. (**B**) Representative images of echocardiography data shown in B- and M-mode images. (**C**) The outcome of GHRP-6 treatment is reflected in the prevention of ventricular dilation and improvement in myocardial mechanical function. All the values are expressed as mean ± SEM; *n* ≥ 6 for each group. * *p* < 0.05, ** *p* < 0.01, *** *p* < 0.001 versus sham; # *p* < 0.05, ## *p* < 0.01, ### *p* < 0.001 versus MI. Group differences were assessed using one-way ANOVA for continuous variables with homogeneity of variance and Brown–Forsythe and Welch’s ANOVA tests for heteroscedasticity data. Nonparametric analyses were performed using the Kruskal–Wallis test for data that do not meet normality.

Seven days after the induction of the AMI, a severe left ventricle dysfunction was detected in saline-treated rats relative to the echocardiographic recordings derived from the sham ischemic controls. Saline-treated animals exhibited a significant decrease in LVEF (47.9 ± 14.9% versus 81.0 ± 2.8%, *p* = 0.0061), LVFS (25.6 ± 9.2% versus 51.4 ± 3.1%, *p* < 0.0001), CO (58.6 ± 17.5 mL/min versus 90.3 ± 13.5 mL/min, *p* = 0.0002), and SV (155.6 ± 42.0 μL versus 246.6 ± 33.0 μL, *p* < 0.0001) (Figure 3A,B). Correspondingly, there was an increase in D, s (5.9 ± 1.1 mm versus 3.7 ± 0.2 mm, *p* < 0.0001) and V, s (178.7 ± 82.8 μL versus 57.3 ± 8.6 μL, *p* < 0.0001). No changes in heart rate were observed.

The 7-day GHRP-6 treatment at the selected dose of 0.4 mg/kg significantly ameliorated left ventricle systolic dysfunction. As compared to saline administration, the treatment increased LVEF (68.1 ± 8.2% versus 47.9 ± 14.9%, *p* = 0.0444), LVFS (39.8 ± 6.8% versus 25.6 ± 9.2%, *p* = 0.0009), and SV (207.7 ± 12.3 μL versus 155.6 ± 42.0 μL, *p* = 0.009). Concomitantly, a decrease in D, s (4.6 ± 0.7 mm versus 5.9 ± 1.1 mm, *p* = 0.0073) and V, s (100.6 ± 35.8 μL versus 178.7 ± 82.8 μL, *p* = 0.0094) was observed (Figure 3A,B).

**Figure 4:**
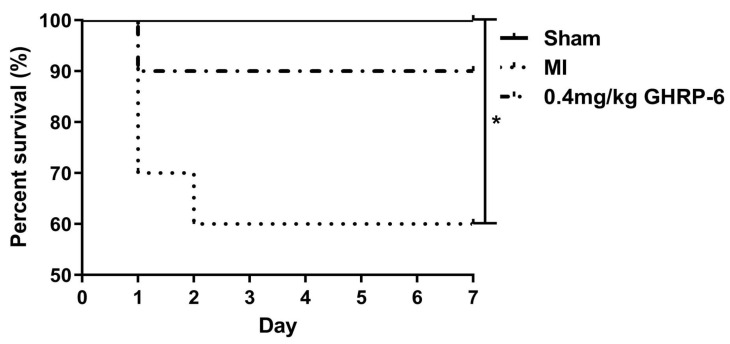
Survival curves of rats in experimental groups; *n* ≥ 6 for each group. * *p* < 0.05 represents difference between infarct + saline-treated rats and the sham ischemic group. Analysis through log-rank tests. Log-rank test was used for Kaplan–Meier survival analysis.

Although the survival curves show a mortality rate of 40% for the saline-infarcted control animals versus a 10% for the GHRP-6 (0.4 mg/kg) intervention group, no statistically significant difference was observed (*p* = 0.0614). No mortality was observed in the sham ischemic group (Figure 4).

**Figure 5:**
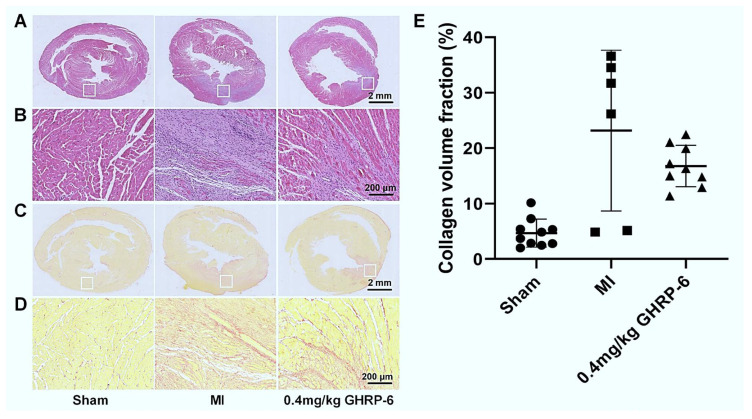
GHRP-6 attenuated myocardial damages and ensuing interstitial fibrosis, according to HE and picrosirius red staining. Left panel: representative image of sham ischemic hearts. Central panel: representative image of infarcted animals receiving saline solution. Right panel: representative image of infarcted animals receiving GHRP-6. (**A**) Representative image of whole heart via H/E staining (scale bar: 2 mm). (**B**) The enlarged image indicated by white squares from A (scale bar: 200 μm). (**C**) Representative image of whole heart via picrosirius red staining. Interstitial collagen material accumulation is stained in red (scale bar: 2 mm). (**D**) The enlarged image indicated by white squares from (**A**) (scale bar: 200 μm). (**E**) Quantitative data for the collagen volume fraction. All the values are expressed as mean ± SEM; *n* ≥ 6 for each group. Group differences were assessed using one-way ANOVA for continuous variables with homogeneity of variance and Brown–Forsythe and Welch’s ANOVA tests for heteroscedasticity data. Nonparametric analyses were performed using the Kruskal–Wallis test for data that do not meet normality.

The analysis of H/E-stained slides revealed substantial qualitative histopathological differences in the myocardium-affected regions between the saline- and GHRP-6 treated group samples. Specimens from the saline control group exhibited extensive interspersed coagulative necrosis, characterized by loss of eosinophilia, cardiomyocyte fragmentation, and lysis, accompanied by marked transitional inflammatory infiltration. Dispersed foci of dense and compact granulation tissue containing secreting fibroblasts and wavy, eosin-stained collagen-like fibers were also observed (Figure 5A,B—locally magnified areas of injury). GHRP-6 appeared to limit the infiltration of inflammatory cells and reduce the extension of dead tissue, consequently reducing the magnitude of accumulated granulation tissue (Figure 5A,B). No histopathological damages were found in the sham-ischemic animals. Post-infarct myocardial interstitial fibrosis measurements using collagen-specific staining indicate that GHRP-6 intervention reduced tissue injury and the consequent scarring, although these differences did not reach statistical significance (Figure 5E). A 27.6% reduction in scarring was observed with GHRP-6 treatment (Figure 5C–E).

### 2.3. GHRP-6 Administration Upregulated the Expression of Myocardial Salvage-Related Proteins/Pathways

**Figure 6:**
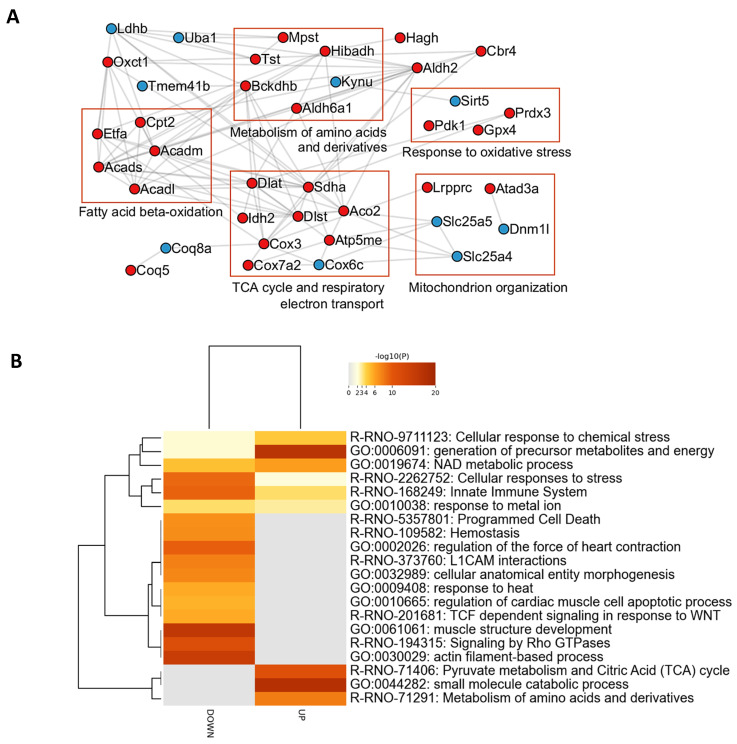
Myocardial proteins and pathways acutely modified by GHRP-6 administration. (**A**) Interaction network among mitochondrial proteins differentially modulated in cardiomyocytes of rats treated with GHRP-6. Proteins are represented according to their abundance levels (blue, decrease; red, increase) in the proteomic profile. Interactions were obtained using the Metascape gene annotation and analysis resource (https://metascape.org/). (**B**) Enrichment analysis for differentially modulated proteins in heart tissue of rats treated with GHRP-6. Biological processes and pathways significantly represented in the proteomic profiles (*p*-value < 0.01, enrichment factor > 1.5) were identified using the Metascape gene annotation and analysis resource (https://metascape.org/). In the heatmap, enriched terms are colored according to *p*-values.

The proteomic study revealed 191 differentially modulated proteins in LV tissue upon GHRP-6 administration (Supplementary Table S1). The GHRP-6-regulated proteome includes 57 proteins (30%) which are located in mitochondria, according to UniProtKB database annotations (Supplementary Table S1). Using such a subset of mitochondrial proteins, we performed interaction network analysis (Figure 6A). Importantly, an array of mitochondrial proteins related to fatty acid beta-oxidation appeared to be upregulated after GHRP-6 treatment. In line with the upregulation of fatty acid beta-oxidation related proteins, GHRP-6 also increases the levels of mitochondrial proteins related to the tricarboxylic acid cycle (TCA) cycle and respiratory electron transport chain (Figure 6A). Furthermore, the mitochondrial pyruvate dehydrogenase kinase isozyme 1 (PDK1), which is an essential regulator of aerobic respiration, was upregulated in response to GHRP-6 treatment. Conversely, glycolytic enzymes (GAPDH, PFK-M, ENO3) and L-lactate dehydrogenase B chain (LDH-B), which prompts pyruvate fermentation to lactate, were downregulated in response to GHRP-6 treatment (Supplementary Table S1). Of note, proteins associated with mitochondrial organization were also differentially modulated in response to GHRP-6 (Figure 6A). GHRP-6 administration enhanced the expression of the ATPase family AAA domain-containing protein 3 (Atad3a) and the mitochondrial transcriptional regulator leucine-rich PPR motif-containing protein (Lrpprc), involved in the metabolic remodeling of mitochondria. Myocardial salvage-related proteins also appeared modulated in response to GHRP-6 administration. Specifically, the generation of precursor metabolites and energy synthesis, pyruvate metabolism, the citric acid cycle, and NAD generation appeared as significantly represented processes in the proteome (Figure 6B; Supplementary Table S2). Furthermore, the GHRP-6-regulated proteome is functionally associated with cellular response to stress (Figure 6A,B). The mitochondrial antioxidant enzymes peroxiredoxin-3 (Prdx3) and glutathione peroxidase 4 (Gpx4) were increased in response to GHRP-6 treatment. Conversely, GHRP-6 downregulated proteins related to cell death (Figure 6B). Finally, proteins related to actin filament-based processes, myocardial contractility, and those involved in the Rho GTPases signaling pathways were downregulated (Figure 6B).

## 3. Discussion

This study investigated the cardioprotective potential of GHRP-6 within the specific context of a permanent coronary ligation (PL) model, a setting that excludes reperfusion and leads to extensive ventricular remodeling. While the salutary effects of GHS family members, including ghrelin, GHRP-2, hexarelin, and GHRP-6, are well-documented in ischemia/reperfusion (I/R) models [17,21,22,23], their efficacy in the clinically relevant but pharmacologically challenging scenario of permanent ischemia remains less explored [16,17,20,24,25].

The model used here is based on a permanent ligation without reperfusion, which simulates extensive left ventricular wall thinning and spherical change. By excluding reperfusion-related factors, it provides an ideal context for evaluating the long-term effects of drugs such as GHRP-6 on myocardial fibrosis, ventricular remodeling, and contractile dysfunction [26]. Furthermore, the GHRP-6 dose selected for the post-myocardial infarct, as for the mitochondrial proteomic studies, derived from the echocardiographic LV functional assessment described here, was adopted from our previous work. In those studies, GHRP-6 at 400 μg/kg was shown to preserve myocardial tissue against lethal injury induced by acute ischemia [17] and anthracycline-induced cardiotoxicity [27]. GHRP-6 was intraperitoneally administered [28] immediately after the ischemic insult with the intent of blunting the acute elevation of cardiac sympathetic nerve activity and the ensuing neurohormone production, as previously documented [24,29,30].

Our findings demonstrate that post-infarct administration of GHRP-6 attenuates adverse ventricular remodeling and systolic dysfunction. Notably, although limited by sample size (*p* = 0.0614), a strong trend towards reduced mortality (10% versus 40% in controls) was observed, suggesting a potential survival benefit. These results poise GHRP-6 as a candidate not merely for myocardial salvage—which the PL model precludes—but for mitigating the subsequent pathological remodeling process. The following discussion interprets these functional and structural improvements in light of the observed molecular shifts, particularly those involving mitochondrial efficiency, to elucidate the potential mechanisms by which GHRP-6 confers protection in the absence of reperfusion.

Irrespective of the compelling evidence documenting that GHRP-6 administration elicits an acute myocardial inotropic activity in healthy rats, measurable through morphologic and functional echocardiographic parameters [31], there is a historical dose range dispersion within the GHS family members across the cardiovascular experimental studies [15,16,17,32]. For obvious reasons we aimed to examine the acute effects of a single administration of three different GHRP-6 dose levels and to select a definitive working dose. As mentioned above, 0.4 mg/kg was the only dose that simultaneously increased all the myocardial contraction-related variables, including stroke volume, suggesting an ample and uniform contractile spike [33]. This dose had previously been shown to significantly reduce infarct size in a porcine model of acute myocardial infarction [17], and it represents the doubling of the dose used in a double-hit cardiac damage model for preventing sudden death in dogs with dilated cardiomyopathy and subsequent acute ischemia [16]. The dose of 0.8 mg/kg was excluded, as it induced bradycardia and systolic volume reduction. Conclusively, although doses, methods, and models to examine a cardioprotective substance at the preclinical level are of major significance, only recently has a comprehensive standardization program come to light [34]. This has remained as drawback for the introduction of GHRP-6 and other cardioprotective candidates in the clinical scenario. 

This non-reperfusion model proved to be a reliable experimental system. After seven days of coronary ligation, heart failure had already been established as judged by the reduction in all the systolic parameters and the concomitant increase in left ventricular systolic volume and diameter, which are all suggestive of an ongoing myocardial remodeling process. GHRP-6 administered during this period attenuated the post-infarct ventricular dysfunction by improving contraction with an increased ejected volume and a reduction in systolic diameter. We deem that this myocardial functional improvement and the amelioration of a ventricular remodeling event contributed, although not significantly, to ensuring survival among the GHRP-6-treated rats. Regardless of the differences in the experimental methodologies across the literature, our data converge to indicate that GHRPs, including GHRP-6, are endowed with the following: (i) positive inotropic effects, (ii) cardioprotective properties in terms of myocardial cells salvaged before otherwise lethal outcomes, and (iii) the ability to improve ventricular systolic function and ameliorate remodeling after myocardial infarct [15,16,22,35]. Similarly, anti-inflammatory and anti-oxidant pharmacologic effects are also described for this family of GH secretagogues [17,32,36,37].

The qualitative observation in the present study of a minor area of damage and smaller granulation tissue accumulation emphasizes previous findings about the GHRP-6-mediated cardioprotection in a porcine model of myocardial infarct based on I/R [17]. The reproducibility of these findings is particularly relevant given the different pathogenic implications of each experimental model. This study employs a non-reperfusion model in which tissue damage is far more severe with larger infarct size, progressive ventricular functional deterioration, rampant remodeling events, and in which infarct size reduction may not be manipulated, given the lack of reperfusion to the area at risk [23,38]. Although the decrease in the collagen volume fraction did not reach statistical significance, an average decreasing trend of approximately 27.6% was observed. The improvement in pathological morphology is highly consistent with the significant cardiac function recovery and the reduced ventricular dilation observed in the GHRP-6 treatment group, which supports the effective inhibition by GHRP-6 of adverse ventricular remodeling after myocardial infarction. Although we did not directly perform morphological quantification of myocardial cell hypertrophy, which is a limitation of this study, future research can adopt more accurate 3D imaging techniques covering multiple levels of the entire heart or more sensitive biomarkers and expand the sample size to more reliably confirm its anti-fibrotic effect. However, establishing whether this reduction in myocardial fibrosis is due to a timely activation of GHRP-6-mediated cardiomyocyte survival mechanisms or derives from the GHRP-6 anti-fibrotic effect is indefinable at the moment and warrants further studies. Both GHRP-6 and ghrelin may rescue jeopardized cardiac cells [10,39] and prevent and remove cardiac and extra-cardiac tissue fibrotic accumulation through different signaling pathways [40,41,42]. Irrespective of what the proximal driver is, it is noteworthy that the distal outcome observed is compatible with myocardial tissue damage reduction. Myocardial infarct size reduction remains as the most robust end point of cardioprotection studies [43]. This study primarily focuses on interstitial fibrosis and overall ventricular geometric changes but does not quantitatively assess myocardial cell hypertrophy—an important structural remodeling indicator (e.g., measurement of myocardial cell cross-sectional area). This represents a limitation of the current study, and future work will incorporate detailed morphometric analysis at the myocardial cell level to more comprehensively elucidate the effects of GHRP-6 on ventricular remodeling.

Furthermore, the positive inotropic effect of GHRP-6 requires contextual interpretation within myocardial ischemia. While increased contractility could theoretically raise oxygen demand and worsen injury, our findings in a permanent ligation model show a net beneficial outcome: GHRP-6 improved systolic function (LVEF, LVFS, SV), attenuated ventricular dilation, and showed a favorable survival trend. This suggests that its cardioprotective mechanisms outweigh any potential detriment from increased contractility. Our proteomic data provide a plausible explanation: GHRP-6 upregulated mitochondrial fatty acid β-oxidation and TCA cycle proteins, promoting more efficient aerobic energy production. This metabolic shift likely enhances energy yield per oxygen consumed, offsetting the oxygen cost of increased work and improving the myocardial oxygen balance. Additionally, the downregulation of glycolytic enzymes and LDH-B indicates reduced anaerobic metabolism, which may lessen acidosis and support diastolic function. Thus, the observed inotropic effect is likely one facet of a coordinated adaptive response, driven by improved metabolic efficiency, that collectively mitigates post-infarct remodeling.

To directly investigate this potential improvement in metabolic efficiency at the molecular level and to gain further insights into the impact of GHRP-6 on mitochondrial physiology, a proteomic study was conducted in healthy heart samples 6 h after peptide administration. This study was prompted by two observations from our group: (I) Mitochondrial preservation represents a key cardioprotective mechanism of GHRP-6 in doxorubicin-intoxicated rats; (II) In cultured H9c2 cardiomyocytes, GHRP-6 administration induces rapid translocation of its receptor CD36 to the mitochondrial outer membrane. Mounting evidence justifies the reason why mitochondria are considered at the epicenter of cardiovascular physiology and pathology [44]. Our study determined that out of 191 proteins differentially modulated by GHRP-6, 57 (30%) are located in the mitochondria. The functional interpretation of the most significantly modulated proteins reveals that GHRP-6 may facilitate fatty acid beta-oxidation and the acquisition of energy precursors, anti-oxidant defenses, cytoprotection by downregulating apoptotic drivers, and mitochondrial structural organization. However, glycolytic enzymes, including lactate dehydrogenase, were downregulated. The identification of these proteins/pathways and their concerted action within mitochondria contribute to shed light on the molecular drivers behind the GHRP-6 cytoprotective abilities. Considering that a human heart uses approximately 6 to 30 kg of ATP per day [45,46] and that mitochondrial bioenergetic conservation is critical for cardiomyocytes homeostasis and survival, it may be relevant that GHRP-6 favors pathways involved in precursor substrates and energy synthesis, which may enable metabolic flexibility and facilitate mitochondrial metabolic reprogramming during ischemia [47].

Altogether, these data support the notion that these therapeutic bounties are driven by the agonistic stimulation of myocardial CD36 and/or GHSR1a receptors, as both may trigger survival pathways and optimize energetic homeostasis shunts [48,49]. The primary mediatory function of these two receptors in cardiac and extra-cardiac organ protection has been controversial and is beyond the scope of our study. However, this is a pharmacologically relevant issue that deserves comment. A line of evidence based on cardiac and non-cardiac cells in which GHS-R1a activation has been silenced concludes that this receptor is irreplaceable for the activation of salvage kinases, e.g., for the anti-fibrotic, anti-oxidant, anti-inflammatory, and proangiogenic effects achieved upon its occupation [50,51,52,53,54]. However, the early observation by Baldanzi et al. in which both ghrelin and des-acyl ghrelin inhibited apoptosis in H9c2 cardiomyocytes, which do not express GHSR1a [55], inaugurated the alternative line, supporting the belief that prosurvival pathways may be triggered and functionally activated through a GHSR-independent pathway. The potential contribution of CD36, in light of our findings, demands consideration and may not be disregarded. First, GHRP-6 binds to CD36; second, CD36 plays a key role in providing the myocardium with its major energy substrate; third, the agonistic binding of CD36 protects against myocardial damage and dysfunction by ischemia/reperfusion; fourth, mice deficient in CD36 exhibit reduced tolerance to myocardial ischemia/reperfusion injury, whereas humans harboring inborn CD36 mutations exhibit a variety of heart diseases; and fifth, like GHSR-1a, CD36 is expressed by endothelial and cardiac muscle cells [49].

This study employed a single endpoint assessment at 7 days post-permanent ligation. While the absence of intermediate time points precludes a detailed kinetic analysis of injury progression and limits our understanding of the temporal dynamics of GHRP-6’s inotropic effect, this design was based on the established pathophysiology of the permanent infarction model, in which a substantial, irreversible infarct is formed within 24–48 h. Our primary objective was to evaluate the efficacy of GHRP-6 in mitigating post-infarct maladaptive remodeling—such as ventricular dilation and systolic dysfunction—rather than acute myocardial salvage. The 7-day time point is a conventional and relevant window in rodent studies for assessing early remodeling, and the significant improvements observed in these end-stage outcomes provide robust evidence for GHRP-6’s beneficial role. We also acknowledge the lack of comprehensive cardio–pulmonary and systemic hemodynamic monitoring post-AMI, which could further elucidate the physiological impact of treatment.

Although growth hormone secretagogues (GHS) have demonstrated a broad spectrum of therapeutic benefits—even for understudied conditions such as anorexia–cachexia syndrome—clinical progress has been modest, yet the reported clinical outcomes remain highly promising [56]. Further safety and mechanistic studies await in order to ensure its broad and successful clinical development [10]. 

## 4. Materials and Methods

### 4.1. Animals and Ethics

The studies on the influence of GHRP-6 dose on the myocardial inotropic effect and the outcomes of AMI were conducted at the Shanghai Institute of Materia Medica, China. Animals were obtained from the Shanghai Center of Experimental Animals. Myocardial tissue mitochondrial proteomic analysis (MPA) was conducted at the Center for Genetic Engineering and Biotechnology (CIGB), and the rats were purchased from the National Center for Laboratory Animals Breeding (CENPALAB, Havana, Cuba). All these studies were conducted using male Wistar rats of 270–310 g of body weight at 10–11 weeks age; maintained in certified rooms with light and temperature-controlled conditions, with food and water available ad libitum; and acclimated for 7 to 10 days before experiment initiation. All animal experiments complied with the international standards stated in the Guide for the Care and Use of Laboratory Animals. The dose–response and the AMI protocols were approved by the Institutional Animal Care and Use Committee at Shanghai Institute of Materia Medica (IACUC number: 2023-10-GDA-109, approved on 27 October 2023). Animal manipulation and investigational procedures for the MPA protocol were approved by the CIGB Institutional Animal Care and Welfare Committee (Protocol No. 027/2022, approved on 7 September 2022). Animals selected for autopsy were terminated by anesthesia overdose (250 mg/kg sodium pentobarbital).

### 4.2. Reagents and Treatments

Isoflurane was purchased from Qingdao Orbiepharm Co., Ltd. (Qingdao, China). Zoletil-50 was obtained from Shanghai Xintu Trading Co., Ltd. (Shanghai, China). Picrocsirius red staining solution was purchased from Shanghai Guduo Biotechnology Co., Ltd. (Shanghai, China). Hematoxylin/eosin staining solution was obtained from Wuhan Boster Biological Engineering Co., Ltd. (Wuhan, China). Ketamine, xylazine, and pentobarbital were obtained from AICA laboratories, BioCubaFarma (Havana, Cuba). The hexapeptide GHRP-6 (His-d-Trp-Ala-Trp-d-Phe-Lys-NH2) was purchased from BCN Peptides (Barcelona, Spain). Solutions were always freshly prepared and protected from light. For the AMI and MPA studies, GHRP-6 was intraperitoneally administered at the selected dose of 400 µg/kg, according to previous findings [17], which were subsequently confirmed in the present dose–response study.

### 4.3. Echocardiography Studies

Anesthesia was induced with isoflurane 4% in 100% oxygen in an induction chamber. The rats were placed supine on a heated fixed plate at 37 °C and provided 1.5% isoflurane in 100% oxygen through a mask covering the nose [57]. Transthoracic echocardiography recordings were performed. The effects of different GHRP-6 doses on post-AMI systolic function were assessed using a Vevo 3100 super-resolution multimodal ultrasound imaging system. (Fujifilm VisualSonics, Inc., Toronto, ON, Canada), focusing on myocardial functional and structural parameters. Structural data recorded included left ventricle diastolic and systolic dimensions (D, d) and (D, s) respectively. Functional parameters included diastolic volume (V, d), systolic volume (V, s), left ventricular fractional shortening (LVFS), left ventricular ejection fraction (LVEF), cardiac output (CO), and stroke volume (SV). Statistical analysis was performed on the echocardiographic images using the data processing software VevoLab (version 5.11, FUJIFILM VisualSonics). LVEF was considered the main functional parameter, as previously indicated [58]. In this study, the echocardiographic data processing was independently performed by a research assistant who was unaware of the group allocation. This individual was solely responsible for standardized measurements and parameter calculations from the echocardiographic images and did not participate in group assignment, drug administration, or surgical procedures.

### 4.4. Experimental Protocols and Study Groups

#### 4.4.1. Protocol I: Dose Response to GHRP-6: Inotropic Effect

Prior to examining the effect of GHRP-6 on AMI-associated ventricular dysfunction, we conducted a pivotal experiment to determine the dose at which an inotropic effect was achieved. Inotropism-related parameters were selected to assess myocardial functional responses, based on established criteria from prior studies [31]. For this purpose, 40 rats were randomly assigned into four groups (*n* = 10/group): (I) saline control, (II) GHRP-6 at 0.15 mg/kg, (III) GHRP-6 at 0.4 mg/kg, and (IV) GHRP-6 at 0.8 mg/kg. Echocardiography recordings were obtained, as described above. Baseline data were registered once the animals were sedated before GHRP-6 administration and 5 min after its administration. 

#### 4.4.2. Protocol II: Effect of GHRP-6 on the Post-AMI Myocardial Status

For the non-reperfusion AMI induction, rats were anesthetized with an intraperitoneal administration of Zoletil-50 (50 mg/kg) and subsequently connected to an ALC-V9 ventilator (Shanghai ALCBio, Shanghai, China). The surgical area was clipped and locally cleansed with povidone-iodine solution. The heart was exposed via a left thoracotomy at the fourth intercostal space, followed by a permanent ligation below the left descending coronary artery, as described in [59]. Regional ventricular ischemia was confirmed by visual myocardial bleaching of the anterior wall of the left ventricle. Sham ischemia was implemented using an identical surgical procedure, except without coronary artery ligation. Before extubation, the lungs were deflated by placing the outlet tube connected to the endotracheal tube in an underwater seal. A total of 30 rats were randomly assigned into three groups (*n* = 10/group): (1) sham ischemic rats receiving normal saline, (2) saline-control infarcted rats, and (3) GHRP-6 0.4 mg/kg for AMI rats. Saline and GHRP-6 were intraperitoneally administered immediately after the surgery, continuing once a day for the following 7 days. The impact of GHRP-6 intervention was evaluated via echocardiographic and histological procedures. Animals were euthanized by anesthesia overdose at the end of the administration phase. Autopsy was performed after anesthesia overdose, and the hearts were extracted and washed in ice-cold normal saline.

### 4.5. Histopathological Analysis of Myocardial Infarct Scar Staining

Rat hearts were sliced, fixed in buffered formalin, paraffin-processed, and serially sectioned at 4 μm. Tissue rims included the macroscopically damaged and normal surrounding area. In addition to hematoxylin/eosin (H/E) slides, samples were processed in picrosirius red solution for 1 h for myocardial fibrosis staining and subsequent analysis, as previously described [60]. The latter is the standard method for evaluating the presence and organization of tissue collagen. Collagen volume fraction was morphometrically determined and expressed as the percentage of the area occupied by collagen divided by the area of the left ventricle, as described in [61]. Photomicrographs were taken using an Olympus BX51 microscope (Olympus Corporation, Tokyo, Japan). Software Image Pro Plus version 6.0 was used for quantitative histological analysis.

### 4.6. Protocol III: Myocardial Tissue Mitochondrial Proteomic Analysis (MPA)

Tissue sample preparation and subcellular fractionation: Myocardial tissue for proteomic analysis was collected from a total of 12 male Wistar rats. The rats were randomly divided into a treatment group and a control group. The treatment group was intraperitoneally administered GHRP-6 (0.4 mg/kg), whereas the concurrent control group received the same volume of normal saline solution (NaCl 0.9%). After a 6 h period, the animals were terminated, and samples of the left ventricle were extracted. Tissue homogenization and differential centrifugation to isolate the mitochondrial fraction were performed as described in the protocols in [62,63]. Protein extraction and sample preparation: To disrupt the mitochondria, the mitochondrial pellet was re-suspended in a lysis buffer containing 6 M guanidine-HCl, 10 mM DTT, 5 mM EDTA, 10% glycerol, protease inhibitors, and 90 mM HEPES-NaOH, pH 7.4, followed by three cycles of rapid freezing/thawing. After centrifugation at 16,000 g for 20 min at 37 °C, the fraction enriched in mitochondrial proteins was contained in the supernatant. Protein concentration was determined using the Bradford method (BioRad, Hercules, CA, USA). Proteins (300 µg) were reduced by incubation with 10 mM DTT for 2 h at 37 °C and alkylated for 30 min at 25 °C by the addition of 40 mM IAA. Proteins were then digested at 37 °C by the addition of lysine endopeptidase (1:100, 16 h) and trypsin (1:50, 16 h). For the peptide isotopic labelling, normal (light, L) and deuterated N-acetoxysuccinimide (heavy, H) were used as described in [64]. The peptide mixtures from the control and treated samples were centrifuged at 12,000 rpm for 10 min, and equals amounts from both samples were then mixed. The complex mixture of peptides was desalted by using a minicolumn (4.6 mm × 50 mm) packed with RP-C4, following a published methodology [65].

Mass spectrometry analysis and protein identification: The complex mixture of peptides was fractionated using strong cation-exchange chromatography (SCX), according to a previously described procedure [66]. Each peptide fraction (RH0, RH1, and RH2) was desalted four times, and three technical replicates were analyzed by reversed-phase chromatography in independent LC-MS/MS experiments. Mass spectrometric measurements were obtained using an Orbitrap Velos Pro spectrometer. The spectra were acquired in the *m*/*z* range from 700 to 6000. MS/MS acquisitions were triggered for 2+, 3+, and 4+ charged precursor ions. Peptides were assigned to MS/MS spectra using the MASCOT search engine against the UniProt database. The following parameters were selected: 0.3 Da as the precursor ion mass tolerance, a tryptic search with up to one missed cleavage, a fixed modification of carbamidomethyl-cysteine, and variable modification of oxidized methionine and deamidation of glutamine and asparagine. The error for matching the daughter ions in the MS/MS spectra was 0.3 Da. The false discovery rate (FDR) was set to 1% for peptide and protein identification. Relative quantification of proteins was performed using a previously described method for N-terminal and lysine acetylated peptides [64]. Proteins containing one or more peptides with an H/L ratio higher than 1.5 or lower than 0.67 (1/1.5) were considered as proteins differentially modulated by GHRP-6.

Bioinformatic-driven functional interpretation of proteomic data: The Metascape web-based tool was used to identify biological processes and pathways significantly represented in the CIGB-500-regulated proteome [67]. Specifically, the Metascape Custom Analysis option was selected, the Homo sapiens genome was used as the background, the Gene Ontology and Reactome databases were selected for enrichment analysis [68,69], and downregulated and upregulated proteins were used as separate input datasets for meta-analysis. The protein interaction network was also retrieved using Metascape 6 and visualized using Cytoscape software (v.3.10) [70].

### 4.7. Statistical Analysis

All statistical analyses were performed using GraphPad Prism software (version 9.0). Continuous data are presented as the mean ± standard error of the mean (SEM). The distribution of data sets was evaluated for normality using the Kolmogorov–Smirnov test (applied when *n* ≥ 5). Data were considered normally distributed if the *p*-value from this test exceeded 0.05. For comparisons involving multiple groups, specifically for the collagen volume fraction analysis, we first assessed variance homogeneity using the Brown–Forsythe test. Based on this, the Brown–Forsythe and Welch’s ANOVA tests were employed, followed by post hoc unpaired *t*-tests incorporating Welch’s correction for pairwise comparisons. Survival data were analyzed using the Kaplan–Meier method, and intergroup differences in survival curves were assessed using the log-rank test. Additionally, the goodness-of-fit for normal distribution (via both Kolmogorov–Smirnov and Shapiro–Wilk tests) and the homogeneity of variances (using Brown–Forsythe and Bartlett‘s tests) were verified for all applicable datasets. Throughout the study, a two-sided *p*-value of less than 0.05 was defined as statistically significant for indicating differences between groups.

## 5. Conclusions

This work offers the first evidence of GHRP-6’s cardioprotective effect under a therapeutic administration scheme in a model of permanent myocardial ischemia, LV mechanical functional demise, and cavity remodeling. The accompanying proteomic analysis contributes to the theoretical assumption supporting the molecular mechanisms of the candidate’s cardioprotective effect, particularly in terms of metabolic reprogramming. Together, these findings may provide a foundation for further exploring GHRP-6’s potential as a therapeutic agent for cardiovascular disease. 

## Data Availability

The original contributions presented in this study are included in the article/Supplementary Material. Further inquiries can be directed to the corresponding author.

## Supplementary Materials

The following supporting information can be downloaded at: https://www.mdpi.com/article/10.3390/ph19030468/s1, Table S1: Proteomic profile; Table S2: Modulated proteins.

## Abbreviations

AMI	Acute Myocardial Infarction
LV	Left Ventricle
HF	Heart Failure
GH	Growth Hormone
GHRP	Growth Hormone Releasing Peptide
GHRP-6	GH Releasing Peptide 6
GHS	Growth Hormone Secretagogue
GHSR1a	GH Secretagogue Receptor 1a
CD36	Cluster Differentiation 36
IGF-1	Insulin-Like Growth Factor Type 1
CENPALAB	National Center for Laboratory Animals Breeding
CIGB	Center for Genetic Engineering and Biotechnology
D, d	Diastolic Dimension
S, s	Systolic Dimension
V, d	Diastolic Volume
V, s	Systolic Volume
LVFS	Left Ventricular Fractional Shortening
LVEF	Left Ventricular Ejection Fraction
CO	Cardiac Output
SV	Stroke Volume
ANOVA	Analysis of Variance
ROS	Reactive Oxygen Species
PCI	Percutaneous Coronary Intervention
ECHO	Echocardiography
